# Invasive species denialism: Sorting out facts, beliefs, and definitions

**DOI:** 10.1002/ece3.4588

**Published:** 2018-10-30

**Authors:** Demetrio Boltovskoy, Francisco Sylvester, Esteban M. Paolucci

**Affiliations:** ^1^ IEGEBA (CONICET‐UBA), Facultad de Ciencias Exactas y Naturales Universidad de Buenos Aires Buenos Aires Argentina; ^2^ Consejo Nacional de Investigaciones Científicas y Técnicas Buenos Aires Argentina; ^3^ Instituto para el Estudio de la Biodiversidad de Invertebrados (IEBI), Facultad de Ciencias Naturales Universidad Nacional de Salta Salta Argentina; ^4^ Museo Argentino de Ciencias Naturales “Bernardino Rivadavia” (CONICET‐MACN) Buenos Aires Argentina

**Keywords:** denialism, impact, invasive species, nonindigenous species

## Abstract

In the last decades, thousands of investigations confirmed the detrimental effects of species translocated by man outside of their native ranges (nonindigenous species, or NIS). However, results concluding that many NIS have null, neutral, or positive impacts on the biota and on human interests are as common in the scientific literature as those that point at baneful impacts. Recently, several scholars confronted the stand that origin per se is not a reliable indicator of negative effects, suggesting that such conclusions are the expression of scientific denialism, often led by spurious purposes, and that their numbers are increasing. When assessed in the context of the growing interest in introduced species, the proportion of academic publications claiming that NIS pose no threats to the environment and to social and economic interests is extremely low, and has not increased since 1990. The widely prevailing notion that many NIS are effectively or potentially harmful does not conflict with the fact that most have mixed (negative, neutral, and positive) impacts. When based on solid grounds, reports of positive or neutral impacts should not be labeled as manipulative or misleading unless proven otherwise, even if they may hamper interest in‐ and funding of research and control bioinvasion programs.

## 1 INTRODUCTION

The debate over the issue of how science and society should deal with the threats posed by nonindigenous species (NIS) dates from the early efforts focused on understanding how and why some organisms succeed in taking advantage of the dispersal opportunities of man's increasing mobility around the globe, while others do not (Davis, 2006). Two opposing lines of thought emerged, vividly illustrated by the Davis versus Simberloff, 2011 debate (Davis et al., 2011; Simberloff, 2011). The core of these discussions is whether NIS share traits that differentiate them from species that do not become NIS or invasive (i.e., NIS whose populations grow vigorously in density and/or areal extension enhancing their use of resources and influencing other members of the community or ecosystem), which boils down to the issue if origin is an overarching attribute significantly associated with the odds of NIS becoming more influential and more harmful in the recipient communities than native species. The answer to this controversy has far‐reaching implications, involving paradigms of ecological theory, as well as management approaches and decisions. In academic media, discussions have centered on the search of scientific evidence and produced massive amounts of data, which fostered healthy and productive debate. However, in efforts to discredit scientists who do not align with the tenet that the alien status of NIS per se entails larger impacts, in the last years some scholars suggested that scientific consensus on the detrimental impacts of NIS is overwhelming, and that opponents to this stand are the expression of purposeful denialism of solid scientific facts, often based on spurious motivations. Further, they suggested that these contrarian pieces of information have been growing in the last decades.

A major point at stake in these discussions is the definition of “large impact,” and especially of "harmful impact." With some notable exceptions, often involving plagues of highly valued cultivated or wild plants and animals (e.g., the Colorado potato beetle *Leptinotarsa decemlineata*, the sea lamprey *Petromyzon marinus*, the snail *Pomacea canaliculata*; Jernelöv, 2017; Joshi, 2017), assessing the size and sign of the effect of a NIS is far from straightforward. We therefore deliberately avoid defining these concepts a priori, referring the reader to the discussions below.

In this article, we first assess whether the relative number of purportedly contrarian academic works has effectively increased since 1990. On the basis of an overview of previous results, chiefly meta‐analyses based on tens to hundreds of case studies, we then assess the consistency and degree of scientific consensus on the notion that NIS have stronger and/or more detrimental effects on the environment and on human interests than native species. We further comment on the reliability of the results published, and discuss some potential sources of bias in their conclusions and in their interpretation by subsequent workers. Finally, we outline the implications of academic research on policy, allocation of resources, and management of biological invasions.

## 2 GROWING DENIALISM?

Building upon previous comments by Richardson and Ricciardi (2013), Russell and Blackburn (2017a, b) recently published provocative arguments against what they perceive as an increase of information opposing the vast majority of the scientific literature that demonstrates the disrupting effects of NIS. These claims were swiftly contested by some of the scholars criticized (Briggs, 2017; Crowley, Hinchliffe, Redpath, & McDonald, 2017; Davis & Chew, 2017; Tassin, Thompson, Carroll, & Thomas, 2017) as unfair and unsupported by evidence. In an effort to back Russell and Blackburn's (2017a, b) perception with actual data, Ricciardi and Ryan (2018a) provided a list of 77 contrarian sources (i.e., pieces of information denying the impacts of NIS), about one third of them from the area of academic literature, and a graph showing how the number of such pieces has increased between 1990 and 2016. Again, this article was rebutted, chiefly on the grounds that it interprets legitimate dissent as denialism (Sagoff, 2018), and the rebuttal was responded by the original authors (Ricciardi & Ryan, 2018b).

**Figure 1:**
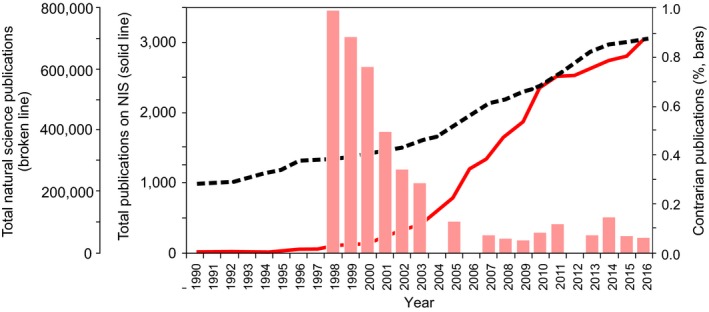
Numbers of academic articles in natural sciences (broken line), and academic publications on nonindigenous species (NIS; solid red line) published between 1990 and 2016 (based on searches of the Scopus database, see text for details), and proportions of publications dismissing the negative impacts of introduced species listed by Ricciardi and Ryan (2018a) (bars, as a proportion of publications shown by the solid red line)

At first glance, the trend illustrated by Ricciardi and Ryan (2018a) is impressive, as the curve it shows climbs dramatically. However, the numbers involved (between 0 and 15 cherry‐picked sources per year) make one frown at their representativeness. A search of the Scopus database (performed on 10 July 2018) using the same terms employed by Ricciardi and Ryan (2018a) (“invasive species,” or “non‐native species,” or “alien species” in the title, abstract or keywords; restricted to the areas Agricultural and Biological Sciences, Environmental Science, Biochemistry, Genetics and Molecular Biology, Earth and Planetary Sciences, and Multidisciplinary, and the years 1990–2016), yielded a total of 27,603 hits. This literature list was downloaded and 159 duplicates were identified and eliminated (using the DOI and title fields). Subsequently, all source names (>95% journals) were scanned for suspicious entries and 18 references unrelated with NIS were deleted, leaving a total of 27,426 works (~98% journal articles, books, and book chapters; Figure 1). Thus, the numbers of academic contrarian pieces listed by Ricciardi and Ryan (2018a) for the same period (1990–2016) represent, on average, ~0.2% of Scopus’ totals (Figure 1).

A similar assessment for the non‐academic sources is more complicated because there are no databases that cover this type of information. However, a Google search of three major websites (YouTube, The New York Times, and Discover Magazine) using the same keywords also shows a growing trend (from 2 in 1990 to 447 in 2016; total 4,229), and the pieces of non‐academic information listed by Ricciardi and Ryan (2018a) account for ~1%. Thus, even if the numbers given by Ricciardi and Ryan (2018a) are underestimating (as are also ours, in particular for the non‐academic information), and all the pieces they cite are effectively contrarian (Crowley et al., 2017; Davis & Chew, 2017; Sagoff, 2018; Tassin et al., 2017), the weight of this denialism is insignificant when compared with the volume of scientific publications on NIS in the same period of time.

Admittedly, we did not check whether the NIS‐related articles identified in Scopus include any that could conceivably be labeled as contrarian. But even if there were some, their numbers would be very low because >95% of the sources involved are from journals in the areas of natural (rather than social) sciences, and therefore are chiefly centered on original scientific research results, rather than on interpretational reviews or philosophical or ethical discussions.

An especially disturbing issue is the fact that the numbers presented by Ricciardi and Ryan (2018a) are given outside of the context of how information on NIS evolved in the period covered. As a matter of fact, this exponential growth of purportedly deceiving information lags behind the also exponential growth in the number of scientific surveys on NIS (Ricciardi & MacIsaac, 2008; Richardson & Ricciardi, 2013). Judging from the list provided by Ricciardi and Ryan (2018a), the proportions of academic contrarian publications show a decreasing (rather than increasing) trend between 1990 and 2016 (Figure 1), making such claims (Ricciardi & Ryan, 2018a; Richardson & Ricciardi, 2013; Russell & Blackburn, 2017a, b) unsubstantiated and misleading.

Incidentally, although interest in invasion biology has clearly increased in the last decades (Canning‐Clode, 2015; Ricciardi & MacIsaac, 2008; Richardson & Ricciardi, 2013), as with contrarian publications, measuring this growth on the basis of absolute numbers of articles is misleading because the total number of papers published in academic journals has been growing by ~4%–5% every year during the last two decades (from 226,626 in 1990, to 699,041 in 2016, according to a Scopus search of all entries in the areas Agricultural and Biological Sciences, Environmental Science, Biochemistry, Genetics and Molecular Biology, and Earth and Planetary Sciences, performed on 10 July 2018; overall total for 1996–2016:11,319,483; Figure 1). The share of NIS‐related academic publications indeed shows a statistically steeper slope than biological output in general (ANCOVA interaction term for year vs. % publications of total for 1990–2016, *p* < 0.001, Figure 1), but the gap is less impressive than when article numbers on NIS are shown outside of this context. As a matter of fact, this relative increase has been particularly strong between ~2000 and 2010, but stabilized thereafter (Figure 1).

## 3 DISSENT, DEBATE, AND DENIALISM

The denialism of solid scientific facts proving that many NIS may—and very often do—have devastating effects on the native flora and fauna is, beyond doubt, harmful for the progress of our understanding of their ecology and their impacts on recipient communities. On the other hand, the assertion that there is ample consensus that NIS pose significant risks to biodiversity and ecosystems is accurate, but it should be clearly distinguished from the lack of consensus that all—or even most—NIS are significant and harmful, which is a matter of intense controversy (Crowley et al., 2017; Davis & Chew, 2017; Davis et al., 2011; Humair, Edwards, Siegrist, & Kueffer, 2014; Jernelöv, 2017; Sagoff, 2005; Simberloff, 2011; Tassin et al., 2017; Young & Larson, 2011).

Dissent can be an expression of honest disagreement (Crowley et al., 2017), or attempts to manufacture conclusions (Ricciardi & Ryan, 2018a), often exploiting the fact that all scientific knowledge contains an element of uncertainty (Russell & Blackburn, 2017b). Both situations occur in the literature on biological introductions, but emphasis on the impacts of NIS, and particularly of those that are perceived as negative, seems more common than their denial, and NIS are often unduly maligned (Brown & Sax, 2004; Humair et al., 2014; Pereyra, 2016). This bias often takes the form of covert hints in the interpretations and discussions of experimental or observational data that show no relationship between origin and impact. In analyzing the density, diversity and composition of epifauna across eight brown seaweed species, including the invasive *Undaria pinnatifida*, Suárez‐Jiménez et al. (2017) concluded that epifaunal traits were unassociated with the origin of the macroalgae, but with the complexity of host morphology, whereby *U. pinnatifida* (and several indigenous species) have simple morphologies and therefore support depauperate epifaunal assemblages. Yet, despite the fact that the area under study (New Zealand) was colonized by this NIS over 30 years earlier, in their concluding remarks the authors note that "abundances of epifauna at the ecosystem level will be reduced if *U. pinnatifida* displaces more structurally complex native seaweed species," omitting the obvious fact that that prospective future dominance by any of the indigenous species with a simple morphology will probably lead to the same result. This type of legitimate, but still lopsided, remarks are hardly surprising, as academic publications, grant submissions, conference reports, newspaper and magazine articles, thesis dissertations, and web pages are unlikely to state that the particular NIS or NIS‐related problem they deal with, or plan to do so, is of little importance to the environment and to society, thus reifying the “native good, alien bad” dichotomy (Goodenough, 2010; Slobodkin, 2001).

Among other possible motivations for dissent, citing Duffy (2013), Ricciardi and Ryan (2018a) mention that “...for some, might be a desire to attain increased visibility; a contrarian message can facilitate exposure in the popular press as well as in some scholarly journals.” Interestingly, the same argument has been used for suggesting that the threats posed by NIS might be inflated. Byers et al. (2002) noticed that “...because positive results are more likely to be submitted and published, the invasion literature may be biased toward demonstrating that nonindigenous species have large ecological impacts.” An evaluation of 651 journal articles published between 1999 and 2014 supported the existence of this bias, but suggested that it has been waning after the mid‐2000s (Warren, King, Tarsa, Haas, & Henderson, 2017).

In academia, many apparent disagreements stem from the fact that the effects of biological introductions are context‐dependent, whereby the impacts are associated with the species and the areas (of origin and recipient) involved (Pantel et al., 2017). Insofar as every NIS represents a new component of an extremely complex and intricate machine (i.e., the community or ecosystem), it invariably has both beneficial and detrimental consequences (as seen from man's perspective), which often change in time (seasonally, multiannually) and space (Atkinson, 1985; Byers et al., 2002; Flory & D'Antonio, 2015; Pearson, Ortega, Eren, & Hierro, 2018; Ricciardi & Whoriskey, 2004; Ruokonen, Karjalainen, & Hämäläinen, 2014; Thomsen et al., 2014). Many species introduced by humans have turned out to be pests or have inflicted economic damage, but one can also make a long list of introductions that have had beneficial effects (Briggs, 2017), and especially of solid, peer‐reviewed reports, that point at significant differences between ecosystems in their response to NIS, of concurrent positive and baneful impacts, and of opposing stands on the sign of their influences (Goodenough, 2010; Sagoff, 2005). For example, the impact of the introduced Argentine ant (*Linepithema humile*) is quite dissimilar over its invasive range, flourishing along the European coasts of the Mediterranean, but with mixed success elsewhere, including Corsica, New Zealand, and the USA (Jernelöv, 2017). Enhancement of toxic cyanobacterial blooms in South America by the freshwater invasive bivalve *Limnoperna fortunei* (Cataldo et al., 2012) is clearly a negative impact which, among many others, is often responsible for massive fish mortalities. However, since its introduction around 1990, the planktonic larvae of this invasive mussel are widely consumed by indigenous fish larvae, for which they represent an abundant, easily available and more nutritious prey than native zooplankton (Paolucci, Thuesen, Cataldo, & Boltovskoy, 2010), and adult mussels are grazed upon by at least 50 South American fish species (Cataldo, 2015), which eliminate up to over 90% of the mussel's yearly production (Duchini, Boltovskoy, & Sylvester, 2018; Sylvester, Boltovskoy, & Cataldo, 2007). The increase in Argentine freshwater fish landings from ~10,000 metric tons in 1950–1990, to ~20,000 tons after 1995, has been tentatively attributed to the presence of this new trophic resource (Boltovskoy, Correa, Cataldo, & Sylvester, 2006). Gauging the balance between these opposite effects and their long‐term impacts on the environment and on social and economic interests is an elusive task, which inevitably involves contradicting opinions when attempting to label the impacts of a NIS (Boltovskoy, 2017), let alone all NIS in toto. Criticisms from both sides rarely target case studies; they are usually centered on attempts at extrapolating case studies to general rules applicable to all bioinvasions, which is the Achilles' heel of invasion science.

## 4 INTERPRETING AND RECONCILING DISSENT

In an effort to resolve or reconcile these disagreements, and exploiting the growing volume of empirical data available, in the last ~15 years several review articles and meta‐analyses using tens to hundreds of case studies were undertaken. They were aimed at pinpointing traits common to NIS, and particularly at elucidating if the overall trends point at impacts different from those of the native species. The issues most frequently addressed were the impacts on abundance, diversity, competition, fitness, and performance, reproduction, growth, survival, biogeochemistry, and nutrient dynamics. In this respect, meta‐analyses employing quantitative methods are conceivably more objective than review articles, because they should be less susceptible to preconceived notions. Although some meta‐analyses concluded that the impacts of NIS are stronger and/or more detrimental than those of indigenous species (Ferlian et al., 2018; Paolucci, MacIsaac, & Ricciardi, 2013; Salo, Korpimaki, Banks, Nordstrom, & Dickman, 2007; Simberloff, Souza, Nuñez, Barrios‐Garcia, & Bunn, 2012; van Hengstum, Hooftman, Oostermeijer, Tienderen, & Mack, 2014; Vilá et al., 2011; Wood et al., 2017; Yoon & Read, 2016), many suggested positive influences and/or that the purported negative effects of NIS are not supported by evidence (Charlebois, Sargent, & Maherali, 2017; Gurevitch & Padilla, 2004; Norkko et al., 2011; Pintor, Byers, & Anderson, 2015; Radville, Gonda‐King, Gómez, Kaplan, & Preisser Evan, 2014; Reise, Olenin, & Thieltges, 2006), and most found variable and context‐dependent impacts (Cameron, Vilà, Cabeza, & Sykes, 2016; Guy‐Haim et al., 2018; Higgins & Vander Zanden, 2010; Howard, Therriault, & Côté, 2017; Martin, Newton, & Bullock, 2017; Nelson et al., 2017; Potgieter et al., 2017; Pysek et al., 2008; Qiu, 2015; Thomsen et al., 2014; Twardochleb, Olden, & Larson, 2013; Vaz et al., 2018; Ward & Ricciardi, 2007), thus hindering broad generalizations.

Several significant points were raised in the literature, especially in review articles, concerning the meaning and the intrinsic, scientific, and social value of “indigenous” versus “invasive,” the concept of “harmful,” the notion and evaluation of “diversity” (Sagoff, 2005, 2018 ; Tassin et al., 2017), and biases in the geographic and taxonomic coverage of the surveys (Pysek et al., 2008; Salo et al., 2007). A particularly sensitive issue is the fact that species with known large and/or negative impacts are selected for investigation far more often than benign species or those considered of little relevance, whereas positive interactions of NIS with natives are under‐reported (Guerin, Martín‐Forés, Sparrow, & Lowe, 2018). The conclusions of review papers and meta‐analyses are subsequently permeated by this bias.

Ricciardi and Ryan (2018a) noticed that most of the academic contrarian articles come from social scientists and philosophers. We acknowledge that these scholars likely have a limited knowledge of the scientific evidences involved. However, rather than dismissing their opinion, natural scientists would benefit from critically evaluating their input, particularly in the light of how NIS are perceived by scientists, managers, and the general public (Humair et al., 2014; Peretti, 1998; van der Wal, Fischer, Selge, & Larson, 2015).

Another source of dissent may stem from semantics, from adherence to a particular belief, and from the abundance and conspicuousness of some invasive species in the recipient area, rather than from conflicting scientific evidence. Attempts at unifying terminology used in research on introduced species (Colautti & MacIsaac, 2004; Davis, 2009; Richardson et al., 2000; Russell & Blackburn, 2017a) have had very limited success (Sagoff, 2018) and explicit opposition (Hodges, 2008; Larson, 2007). Despite (or because of) its value‐laden implications, the term "invasive" has been growing in acceptance (Pereyra, 2016), to the point that the field itself is usually referred to as "invasion biology," even by scholars opposed to the concept that all NIS are harmful (Davis, 2009). “Invasive” is a value‐laden adjective, with unsubtle negative connotations (Davis, 2009; Russell & Blackburn, 2017b), and the notion of “negative” has been incorporated by researchers and international organizations in their definition of “invasive” (IUCN, 2018; Russell & Blackburn, 2017a, b; Simberloff et al., 2012; WWF, 2018). Further, attempts have been made at associating abundance in the recipient area and terminology with social values. Russell and Blackburn (2017a) suggested that the term “invasive” should be applied to the NIS with negative impacts only (but, surprisingly, they also noticed that the impacts of non‐invasive NIS “can be positive and negative, often a combination of both, and **potentially benign overall**”; our emphasis). The distinction between NIS and invasive has traditionally been based on a subjective appreciation of the abundance and impact of the alien species in the recipient environment, whereby NIS are present in moderate numbers, whereas invasives are very abundant. However, as mentioned above, most scholars use "invasive" rather indiscriminately for any NIS (Pereyra, 2016). Thus, implicit or explicit adherence to the notion that "invasive" necessarily involves deleterious impacts, and usage of "invasive" for all introduced species leads to the tautological conclusion that NIS are harmful. We agree that NIS that become very abundant in the recipient range (invasive) are more likely to have large effects than those that do not, but we object the ensuing assumption that large effect equates negative, even though this is often the case.

Dissent on the damage of the impacts is also engendered by the fact that most meta‐analyses center their interest on the net effect sizes of the NIS (usually through exclusion‐inclusion experiments or field observations), while the issue whether the organisms or processes affected involve environmentally or societally negligible (even when statistically significant), negative, or positive changes is given less consideration. For example, Paolucci et al. (2013) performed a meta‐analysis comparing the pressure of indigenous versus NIS consumers on native organisms. They concluded that terrestrial and freshwater (but not marine) NIS have larger effects on native resources than native consumers. This survey was not aimed at assessing the ecological, societal, or economic value of the resources fed upon. As most similar meta‐analyses, the authors' purpose was not investigating whether the species consumed were highly valued plants or animals, or nuisance weeds, or pests. Within this framework, the use of “more negative” (resulting from the algorithm used in this and many other meta‐analyses) for the more voracious introduced consumers and “less negative” for the native ones was meant to identify the ones that are more likely to engender community or ecosystem‐level changes. However, subsequent interpretations of these specific results were used to support statements such as “substantial ecological and economic damage” (Emde et al., 2016), and “non‐native species are more likely to become a pest” (Verbrugge, Leuven, & Zwart, 2016). Change per se is often equated with negative impact, either explicitly or implicitly, entailing that the NIS responsible for the change are deleterious.

Maritime cordgrasses (*Spartina* spp.) are considered “powerful ecological engineers...” (i.e., species that change the character, dynamics, form, or nature of ecosystems over substantial areas; Jones & Lawton, 1994) “...that are highly valued where they are native” (Strong & Ayres, 2013); however, “elsewhere, they overgrow native salt marsh and open intertidal mudflats, diminish biota, increase costs of managing wildlife, and interfere with human uses of estuaries” (Strong & Ayres, 2013). Overgrowth of native vegetation and reduction of biota can, indeed, be interpreted as negative outcomes. Overgrowth of bare mudflats is more debatable (after all, that is what all cordgrasses do, regardless of their origin), but increasing costs of managing wildlife (i.e., eliminating the introduced seagrass), and interfering the human uses of estuaries is clearly contradictory with the fact that they should be protected where they are native (i.e., not wiped out by grazing by cattle or for urbanization). The smooth cordgrass, *Spartina alterniflora*, historically assumed to be an indigenous, highly valued feature of the Atlantic coasts of South America, was suggested to have **catastrophically** (our emphasis) altered the pristine state of nature when its native status was questioned (Bortolus, Carlton, & Schwindt, 2015). Although many arguments on the positive and negative effects of NIS are disputable, some are plainly untenable. The invasive, freshwater bivalves *Corbicula fluminea* and *Limnoperna fortunei* were included in the roster of impacts associated with the depletion of the exploited marine blue mussel, *Mytilus edulis platensis* (Defeo et al., 2013), with which they share neither space nor resources.

## 5 SCIENCE AND POLICY

**Figure 2:**
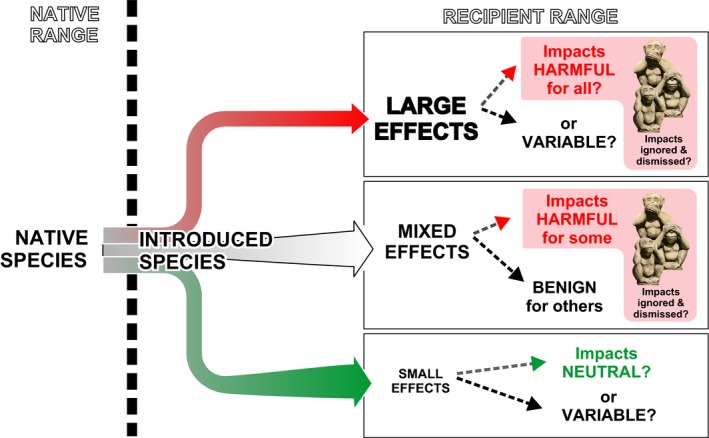
Upon introduction, some species may have large effects on the recipient ecosystems, but these effects are not necessarily all harmful (upper panel). Most introduced species have mixed effects, negative for some resident species or processes and positive for others (middle panel). Many introduced species have no major impacts, which does not entail that their small effects are not favorable for some resident species and deleterious for others. There is no evidence in the scientific literature that the negative effects of NIS are systematically ignored or dismissed (pink background)

Russell and Blackburn (2017a) stated that “negotiating the tensions of perceived consensus alongside scientific uncertainty are critical, especially in the public's eye.” This observation was subsequently echoed by Ricciardi & Ryan's claims that "Effective management of invasive species and other environmental problems requires community consensus,” and scientists should make the scientific consensus known (Ricciardi & Ryan, 2018a). Such statements sound like euphemisms for demanding that, in order to avoid hampering management efforts, researchers should only report the negative effects of NIS. In our opinion, the fact that controversies in the scientific arena can have consequences in the public opinion, and in the funding and implementation of policies oriented at controlling biological invasions (Ricciardi & Ryan, 2018a), should not affect the dissemination of scientifically solid evidences, not only when they suggest negative impacts, but also when they point at neutral or positive effects. Management actions to curtail the spread of NIS are based on a precautionary principle (Russell & Blackburn, 2017b), but scientific conclusions should not be skewed by this legitimate purpose. We agree with Duffy (2013) that “One will need to ignore the possible side effects, as some journalists...” [policy‐makers, managers] “...will inevitably oversimplify one's message and others will twist it to advance a particular political or belief system.” Further, tagging all introductions with the same "harmful species" label may foster policy and management funding and efforts aimed at curtailing the risks of some highly focused introduction pathways, such as ballast water (Bailey, 2015). However, when used indiscriminately and without solid evidences, it may backfire engendering distrust, weakening interest, and decreasing resources for addressing those NIS that have clearly been shown to be most detrimental (Gurevitch & Padilla, 2004; Nentwig, Bacher, Kumschick, Pyšek, & Vilà, 2017). It is estimated that only ~5%–20% of all introduced species become problematic (IUCN, 2018), and around 1%–2% make it to the "worst invasives" lists (European Environmental Agency, 2018; Nentwig et al., 2017), thus deserving prioritization in management efforts (Figure 2). In this respect, crying wolf every time a new NIS is recorded and speculating over the purportedly negative impacts of all NIS can be counterproductive.

## 6 CONCLUSIONS

After several decades of intensive research, the consensus that NIS may engender major changes with negative implications for the biota and for human interests is overwhelming, even among scholars that have been labeled as contrarians (Davis & Chew, 2017). This stance prevails in the academic literature, and it does not seem to have decreased in the last decades. The volume of information denying any negative effects is negligible. Although examples of beneficial NIS are abundant as well (Davis, 2009; Ewel et al., 1999; Jernelöv, 2017), given the hazards involved in each new introduction all efforts possible for keeping introductions from spreading should be undertaken. However, research on the newcomers should neither be tainted by the fact that some previous NIS proved harmful, nor by the fact that some have melted into the ecosystem without major consequences, or even have ended up being beneficial. Every new introduction (and, unfortunately, the trend is on the rise worldwide: Seebens et al., 2017) should be evaluated in its own right and in the particular area invaded (Nelson et al., 2017; Wagner, 1993). The a priori axiom that all NIS are harmful is as detrimental to the buildup of reliable knowledge as is the tenet that NIS are harmless. It is unlikely that either preconceived outlook should influence the outcome of experiments or observations, but their interpretation can be affected, either explicitly or implicitly (Warren et al., 2017).

Our understanding of invasion ecology evolved significantly in the last decades (Simberloff & Vitule, 2013). As in any other field, this maturation involves not only new knowledge and a deeper insight, but also a growing recognition of complexity and ambiguity (Davis, 2009; Davis & Chew, 2017), which in turn engenders healthy and productive debate on the impacts of NIS. Muffling conflicting results on the basis of the argument that they serve spurious purposes does a poor service not only to science, but also to society. We obviously agree that inaccurate, unsubstantiated, deceiving and manipulated results and conclusions in academic and non‐academic media must be confronted. However, exposure of such pieces should apply not only to those that maliciously or simply erroneously dismiss the negative impacts of NIS, but also to the ones that groundlessly inflate their effects. In the words of William Blake (Proverbs of Hell), “*The crow wish'd everything was black, the owl that everything was white*.” The role of science is not stripping nature from the multiple shades of gray, even if black or white were politically more correct and strategically more convenient.

## CONFLICT OF INTEREST

None declared.

## AUTHOR CONTRIBUTION

D.B. conceived the study and wrote the preliminary draft. All authors contributed to analyses of the data, discussions and writing the final version.

## DATA ACCESSIBILITY

No unpublished information was used in this work. All the supporting data are included in the references provided.
